# Environmentally Induced Epigenetic Transgenerational Inheritance and the Weismann Barrier: The Dawn of Neo-Lamarckian Theory

**DOI:** 10.3390/jdb8040028

**Published:** 2020-12-04

**Authors:** Eric E. Nilsson, Millissia Ben Maamar, Michael K. Skinner

**Affiliations:** Center for Reproductive Biology, School of Biological Sciences, Washington State University, Pullman, WA 99164-4236, USA; nilsson@wsu.edu (E.E.N.); millissia.benmaamar@wsu.edu (M.B.M.)

**Keywords:** review, Weismann barrier, Lamarck, Darwin, epigenetics, transgenerational, evolution, sperm, egg

## Abstract

For the past 120 years, the Weismann barrier and associated germ plasm theory of heredity have been a doctrine that has impacted evolutionary biology and our concepts of inheritance through the germline. Although August Weismann in his 1872 book was correct that the sperm and egg were the only cells to transmit molecular information to the subsequent generation, the concept that somatic cells do not impact the germline (i.e., the Weismann barrier) is incorrect. However, the doctrine or dogma of the Weismann barrier still influences many scientific fields and topics. The discovery of epigenetics, and more recently environmentally induced epigenetic transgenerational inheritance of phenotypic variation and pathology, have had significant impacts on evolution theory and medicine today. Environmental epigenetics and the concept of epigenetic transgenerational inheritance refute aspects of the Weismann barrier and require a re-evaluation of both inheritance theory and evolution theory.

## 1. Introduction

August Weismann was an influential biologist of the late 1800s who helped formulate thoughts on heredity and evolution, laying some of the foundation for our current understanding of mechanisms of inheritance. Weismann proposed that only germ cells carry heritable information from one generation to the next [1]. He also proposed the idea of ‘the continuity of the germ line’; the idea that germ cells contain all the necessary information (i.e., germ plasm) to form all the different cells of the next generation, and that this germ plasm information is replicated and kept isolated in the germ line cells (sperm or eggs) as it is passed from generation to generation (Table 1). Therefore, germ cells, when they form embryos, can direct the formation of the somatic cells that make up the rest of an organism, but somatic cells do not pass heritable information to germ plasm. This last point came to be known as the Weismann barrier or Weismann doctrine [2]. Weismann’s theories on germ plasm were presented at a time when the ideas of Darwin and Lamarck were being vigorously debated. Both Darwin [3] and Lamarck [4] had proposed the idea that acquired characteristics in an organism could be inherited. Darwin’s pangenesis theory proposed that the body’s somatic cells produced ‘gemules’ that were small units of heritable information that circulated in the blood and collected in the gonads to form germ cells. Weismann’s ideas were in direct opposition to this, and the experimental evidence of the time seemed to support the idea that somatic cells did not pass to or influence the heritable information in germ cells [5]. Thus, the idea of the Weismann barrier became entrenched in biological thought, and the idea of the inheritance of acquired characteristics has been largely discounted.

Our increasing understanding of the mechanisms of development and evolution in the last decades has uncovered evidence that challenges the presence of a strict Weismann’s barrier. For example, it is now known that germ cells, like all other cell types, undergo developmental programming using epigenetic mechanisms that can influence which genes are turned on or off [6,7,8,9,10,11]. Epigenetic factors are “molecular factors and processes around DNA that regulate genome activity independent of DNA sequence, and that are mitotically stable” [12]. Epigenetic factors include DNA methylation, histone modifications, changes to chromatin structure and expression of non-coding RNAs. Epigenetic developmental programming in germ cells can include changes in DNA methylation [13,14], histone modifications [10,15,16,17], chromatin structure changes [18,19,20], and non-coding RNA changes [21,22,23]. There is some plasticity in these epigenetic changes in germ cells, and this can lead to changes in gene expression in the next generation, even without changes being present in the DNA sequence. Developmental programming in germ cells is heavily regulated by the surrounding somatic cells (Sertoli cells for sperm, and granulosa cells for oocytes) [24,25,26,27,28,29]. So, it can be argued that alterations in these somatic cells (an acquired characteristic) can pass information to germ cells that results in changes in the next generation. Additionally, in mammalian embryos there is a period of development after fertilization during which the embryo is composed primarily of totipotent blastomeres, any of which could become primordial germ cells once the epiblast forms [30]. These blastomeres are somatic cells, so the continuity of the germ line as proposed by Weismann is interrupted by a period of somatic cell development. Changes in somatic cells that become primordial germ cells could certainly influence the germ cells, and again call into question the integrity of the Weismann barrier.

The findings of biological research from recent decades require that we re-evaluate the concept of the Weismann barrier. The current review will describe Weismann’s theories of germ plasm and inheritance, pointing out where he was correct and where he was mistaken. Recent research into the effects of environment on germ cell development and the heritability of those effects will be considered with respect to the Weismann barrier. A re-evaluation of the Weismann barrier can affect how we think about environmental research, disease etiology, and neo-Darwinian evolution theory.

### 1.1. Weismann’s Germ Cell and Inheritance

Weismann’s germ plasm theory of heredity proposed that germ cells were the only cells involved in carrying heritable information to the next generation and forming the embryo [1]. At this time the roles of sperm and egg were still under some debate. However, microscopic studies of sperm and egg development had occurred, as well as studies of early embryo development. Weismann concluded, correctly, that the dark-staining material in the nuclei of developing sperm and eggs (i.e., chromosomes) was the material that carried information to form the cells of the next generation, and that the nuclear material of sperm combined with that of eggs. He even proposed, again correctly, that the heritable material of sperm and eggs must undergo what we now call a reduction division prior to fertilization, so that the amount of heritable material does not double with each generation. In this part of his germ plasm theory of heredity, Table 1, that sperm and egg are the only cells to carry the necessary heritable material to the next generation, Weismann has been proven correct.

### 1.2. Weismann’s Germline (Units of Inheritance Mechanism)

Some aspects of Weismann’s germ plasm theory have been shown to not be accurate. Weismann proposed that molecular ‘determinants’ carried the information needed to specify how cells in a developing embryo would differentiate to become a specific cell type, such as a muscle cell or skin cell. These determinants can be thought of as analogous to genes, although the work of Gregor Mendel had not yet been rediscovered. The collection of all the determinants in the nucleus was called the germplasm in a germ cell, or the somatoplasm in a somatic cell. Weismann proposed that germplasm carried a full set of determinants to instruct the development of every cell in the body. After fertilization, as the cells of the embryo divide, Weismann thought that the germplasm was divided up among the dividing cells, until certain cells had only those determinants needed to specify them to be a particular somatic cell type. These somatic cells would have somatoplasm, containing only a subset of the determinants present in germplasm. With this theory the germ cell lineage would have to be isolated and replicated across generations so that a full set of germplasm was available for each generation’s sperm and eggs, which Weismann termed the continuity of the germ line. In addition, it should be noted that since somatic cells were not thought to have the full complement of ‘determinants’, it was therefore impossible for somatic cells to develop into germ cells, as somatic cells could not produce germ plasm with its complete set of heritable information.

We now know, of course, that somatic cells do have a full complement of genetic material. There are also problems with the idea of the continuity of the germ line. In some species, such as ascarid roundworms, the germ line is determined and identifiable very early after fertilization [31], supporting the idea of the continuity of the germ line. In mammalian species however, after fertilization cleavage divisions produce an embryo composed of blastomeres. These are totipotent or pluripotent somatic cells, any of which could become primordial germ cells later in development [31]. This interrupts the continuity of the germ line with a period of somatic development.

The primordial germ cells (PGCs) that develop from the epiblast and migrate down the genital ridge to colonize the fetal gonad at the time of gonadal sex determination are the germline precursor cells [32]. The PGCs are the stem-like cells for the germline that develops into either the prospermatogonia or oogonia at the onset of gonadal sex determination. These somatic cells generate or initiate the development of the female or male germ cell lineage. The DNA methylation erasure that occurs during PGC migration down the genital ridge allows the PGCs to become the stem-like cells for the germ cell lineages [33]. Therefore, a somatic cell type (i.e., PGCs) generates the germ cell lineage. In addition, embryonic stem cell cultures or induced pluripotent stem cells (IPSC) [34,35] have been shown to develop into either sperm or egg in vitro [34,35]. Therefore, somatic cells can generate the germline in a way that is contrary to the Weismann barrier and the continuity of the germ line.

### 1.3. Weismann’s Germline and Somatic Cell Barrier

As mentioned previously, Weismann’s germ plasm theory proposed that only germ cells carried a full set of heritable instructions, and that somatic cells ended up with only subsets of the ‘determinants’ in germ plasm. As a result, somatic cells cannot contribute to the germ line, as they do not have all the necessary determinants. As time passed after Weismann’s germ plasm theory was introduced, the idea that somatic cells do not contribute to the germline became known as the Weismann barrier. Furthermore, as knowledge of genes, chromosomes and heredity grew, the concept of the Weismann barrier shifted somewhat to the idea that hereditary information moves only from germline cells to somatic cells, and not the reverse. More broadly, the idea is that changes acquired in the somatic cells during an organism’s life cannot affect its offspring.

**Figure 1:**
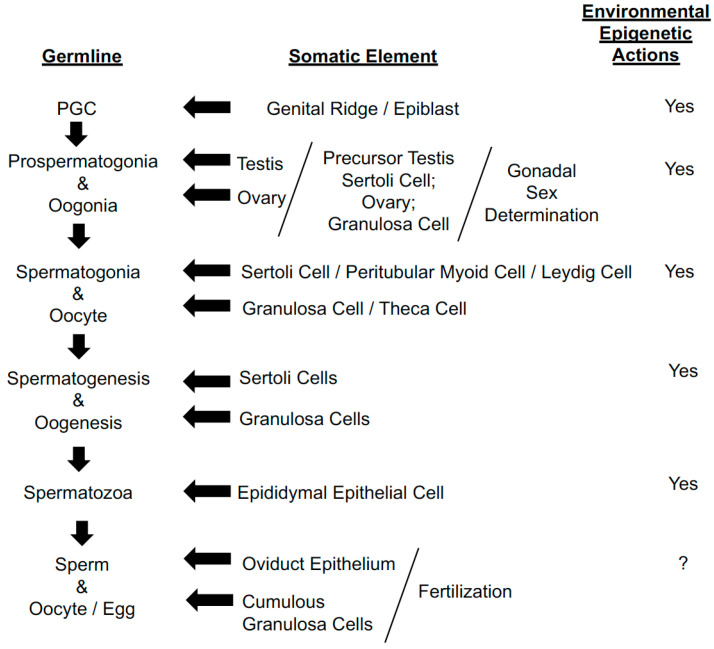
Somatic cell impacts on germ cells. Various germ cell stages from primordial germ cells (PGC) to sperm and egg and the somatic cells that regulate and impact the development and molecular differentiation. The sites of environmental epigenetic regulation are indicated.

In the early 1900s, little was known about the role that somatic cells played in gonad function. We now know that somatic cells regulate germ cells at most every stage of development (Figure 1). Alterations in somatic cell function at any of these stages could result in changes in the germ cells, most likely epigenetic changes, that can result in altered gene expression and altered phenotype in the offspring (Figure 1). Experimental studies have shown that exposure of pregnant rats to environmental factors such as toxicants at the time of gonadal sex determination can result in subsequent F1, F2 and F3 generation offspring with an altered phenotype and increased disease incidence [36,37]. At the time of gonadal sex determination, the primordial germ cells (PGCs) are undergoing a DNA methylation erasure and re-methylation process [38]. Male germ cell re-methylation is dependent on the actions of fetal Sertoli cells [39,40]. While it is possible that the environmental exposures directly affect the F1 generation developing germ cells in the embryo, it is equally reasonable that the exposures (e.g., toxicants) affect the somatic fetal Sertoli cells, which in turn alter DNA methylation in the F1 generation germ cells. This F1 generation germline epigenetic alteration can become permanently programmed to alter phenotypes in the subsequent F3 generations. After ancestral exposure to environmental toxicants, the transgenerational F2 and F3 generation rats have been shown to have epigenetic changes in Sertoli cells and granulosa cells [41,42]. These changes are associated with DNA methylation changes in F3 generation sperm [36]. Therefore, environmental actions on gonadal somatic cells can, on a molecular level through epigenetics, alter the developing germline to impact the subsequent offspring.

Another example of somatic cells affecting germ cells and germ cell function is the process of extracellular vesicles present in the epididymis delivering ncRNA to epididymal sperm, and that sperm carrying the ncRNA to a newly formed zygote at fertilization [43,44,45,46]. Although further research is needed to demonstrate that epididymal ncRNA is actually transferred to the sperm nuclei, and not simply the sperm head, observations suggest this is an additional somatic regulation of the germline. These examples show that there are several times during germ cell development where altered somatic cells can cause epigenetic changes in germ cells (Figure 1), leading to changes in gene expression and phenotype in the next generation, thus violating the concept of the Weismann barrier.

It has now been shown that pluripotent embryonic stem cells in culture can be induced to differentiate into germ cells [47,48,49,50], with accompanying changes in epigenetic reprogramming [51,52]. That these somatic cells can be made to become germ cells also clearly violates the Weismann barrier.

### 1.4. Weismann’s Barrier Summary

August Weismann’s germ plasm theory of heredity was correct in proposing that sperm and eggs are the cells that carry the material of heritable information to the next generation to form the developing embryo. Other parts of the theory were not accurate, such as the continuity of the germ line, and the idea that somatic cells could not contribute heritable information to germ cells (i.e., the Weismann barrier). This is not surprising, since at the end of the 19th century little was known about DNA, cell and developmental biology, or epigenetics. Weismann constructed his theories out of what little he could see of animal development and chromosomes through the microscopes of the time. He incorrectly proposed that germ plasm, with its hereditary determinants, was divided up among the somatic cells of a developing embryo. If one assumes this is true, then somatic cells cannot contribute to the germ line. Thus, the concept of the Weismann barrier is constructed upon faulty assumptions. We now know that somatic cells regulate germ cell development and function throughout the gametogenesis process, and that somatic alterations can result in modified germ cells (Figure 1).

Stem cell studies of the germline PCGs have clearly demonstrated the plasticity of the germ cells [53]. The epigenetic alterations (DNA methylation erasure) that create a stem-like cell and then the re-methylation that occurs to generate the male or female germ cell lineage directly supports the idea that a dynamic developmental process is involved in germ cell development. These observations clearly demonstrate the cellular plasticity of the germ cells and organism, which negates the simplistic view of pre-programmed germ cell theory proposed by Weismann [54].

### 1.5. Epigenetic Transgenerational Inheritance

Epigenetic mechanisms such as DNA methylation and histone modifications provide the ability of the environment to promote cellular plasticity and alterations in organism phenotypes. Environmental epigenetics provides the conduit by which the environmental factors, such as nutrition, temperature, or toxicants, can impact the biology of the organism [12,36]. When the germ cell epigenome is modified through environmental epigenetic actions, then the potential for epigenetic inheritance develops. The germline is now known to be influenced by environmental factors through all stages of development from the primordial stem-like germ cells through gametogenesis to the stages of sperm or egg [6,7,55,56]. When the gamete passes this information to the subsequent generation, this is referred to as epigenetic inheritance. When this inheritance continues in the absence of continued environmental exposures, this is referred to as epigenetic transgenerational inheritance [37,57]. This is a form of non-genetic inheritance that can generate an organism’s plasticity, disease susceptibility, and phenotypic variation [36,58,59,60,61,62,63]. Through this epigenetic transgenerational inheritance process, a gamete with altered epigenetics can alter the epigenetics and transcriptome of the early embryo stem cells, such that all derived somatic cells have an altered epigenome and transcriptome [12,36,41,42,46,64]. The altered somatic cells therefore play a critical role in mediating the environmental epigenetic action on physiology and phenotypic variation in the individual. This epigenetic transgenerational inheritance is the new science that questions many of our previous paradigms in biology.

The Weismann barrier and concepts are challenged by this environmentally induced epigenetic transgenerational inheritance phenomenon. When an environmental exposure can promote generational impacts for hundreds of generations in plants and flies [36,65], as well as other organisms such as worms and mammals [37,66,67,68], our concepts such as cellular predetermination need to be re-evaluated and assessed. Epigenetic transgenerational inheritance is a clear example of the inheritance of acquired characteristics, introducing epigenetic changes into the germline. Germline epigenetic variation appears to promote in the individual and subsequent generations phenotypic variation and the induction of genetic variation in the population [69,70,71]. Therefore, the molecular control of biology as known today needs to be used to re-evaluate the dogmas and concepts developed over the past century in science, such as the Weismann barrier [1]. Clearly August Weismann’s theories in the late 1800s were instrumental toward allowing science to start to understand inheritance mechanisms in the absence of any known molecular information, but it is now time to re-evaluate some of these concepts and dogma, such as the Weismann barrier [32].

### 1.6. Neo-Lamarckian Evolution Theory

**Figure 2:**
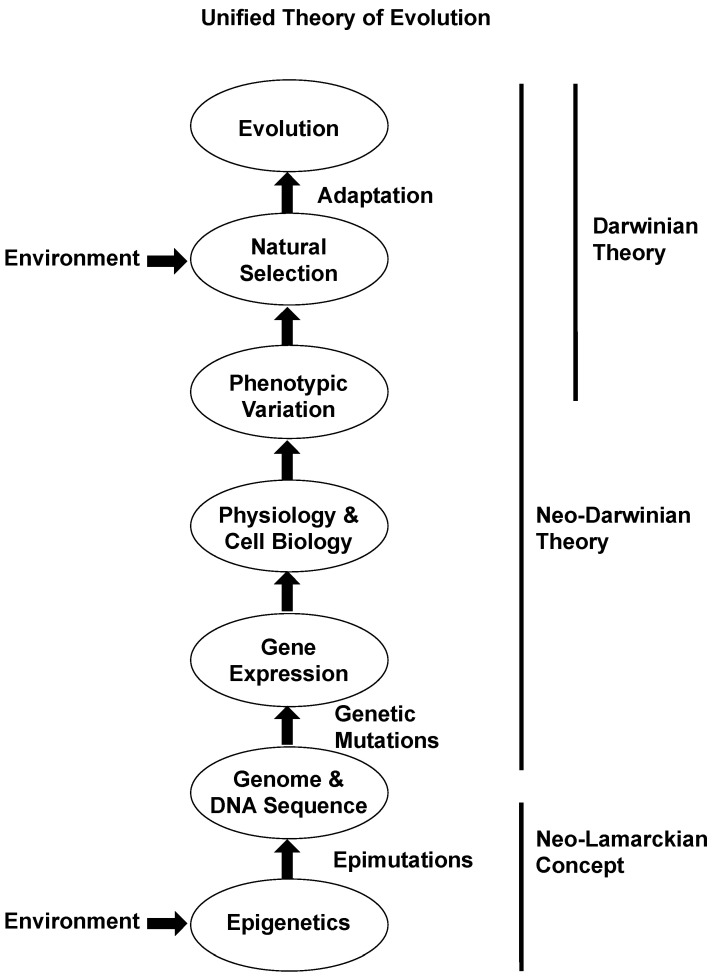
Schematic of the unified theory of evolution. No dominance is suggested by the appearance of specific circles (e.g., epimutations vs. genetics) such that all are equally important components. Modified from [74].

Lamarck proposed in the late 1700s and early 1800s one of the first concepts for evolutionary biology theory “that environment promotes phenotypic variation that becomes heritable” [72]. Although several aspects of Lamarck’s evolution theory were not accurate, this basic concept was the primary observation or mechanism that was put forward by Lamarck. In the 1850s Charles Darwin put forward his theory of evolutionary biology that phenotypic variation acted upon by natural selection allows for adaptation to facilitate evolutionary biology [73]. In the late 1800s Weismann’s theories were used to refute Lamarck’s theories and support some aspects of Darwin’s theory [1]. Weisman’s concept of a pre-programmed germline that was not responsive to environmental impacts became the premise for neo-Darwinian theory in the 1900s along with the incorporation of genetics into our current theory of evolutionary biology [74], Figure 2. We now propose that, while natural selection and genetics are critical for evolution, they are simply not the complete story, nor able to explain some aspects of evolution.

The concepts of environmentally induced epigenetic transgenerational inheritance allow for the “environment to directly impact phenotypic variation and be heritable”, which is the basic concept put forward by Lamarck [4]. Therefore, a neo-Lamarckian concept can augment the genetic mechanisms of neo-Darwinian theory to dramatically impact Darwinian natural selection [74], Figure 2. This provides a more accurate and complete view of the molecular and physiological aspects of evolutionary biology, as well as basic physiology. This does not refute Darwinian theory or the genetics of neo-Darwinian theory, but simply adds new science to facilitate and better understand evolution. Therefore, the use of Weismann’s theory to refute Lamarckian evolutionary theory was not accurate [1], considering our current science, and the incorporation of neo-Lamarckian theory is needed for a better understanding of evolutionary biology [74], Figure 2. The discovery of this non-genetic form of inheritance “environmentally induced epigenetic transgenerational inheritance” promotes a better understanding of biology, inheritance, and evolution. This requires a re-evaluation of the late 1800s concepts of Weismann and current evolution theory.

## 2. Conclusions

The scientific philosopher, Thomas Kuhn, suggested paradigm shifts in science require a generation of scientists to be realized, due to the resistance by the existing generation of scientists to reconsider current dogma [75]. There have been many generations of scientists since the proposal of August Weismann’s theories and even more since Lamarck and Darwin proposed theories of evolution. The progress of science would dramatically increase if we took Kuhn’s advice and critically reviewed and challenged our current scientific paradigms and dogma. Clearly, our current knowledge of germ cell development and biology requires a reconsideration of Weismann’s theories and the dogma of the Weismann barrier. In addition, the discovery of environmentally induced epigenetic transgenerational inheritance of phenotypic variation requires a re-evaluation of Lamarck’s and Darwin’s theories of evolution [74]. This is not a denigration or insult of these previous prominent figures in science, but simply taking note of our current advancements in science and allowing science to progress, rather than be delayed due to the reliance of established dogma.

## Figures and Tables

**Table 1:** Weismann’s Germ Plasm Theory Components.

(1)	Germ cells are the only cells to transmit molecular heredity information between generations.
(2)	Germ cells are the only cells with a full set of instructions (germplasm) for development of the next generation. The determinants of germ plasm are divided up among the somatic cells of the embryo. The full set of instructions is kept intact by germ cells from generation to generation by the continuity of the germ line.
(3)	Germ cell molecular determinants are not impacted by changes in somatic cells (germ line—somatic barrier).
